# Herpes zoster of the trigeminal nerve with multi-dermatomal involvement: a case report of an unusual presentation

**DOI:** 10.1186/s12895-020-00110-1

**Published:** 2020-10-30

**Authors:** Lorenzo Stefano Pelloni, Raffaele Pelloni, Luca Borradori

**Affiliations:** 1 Department of Dermatology, University Hospital of Bern, Inselspital, Bern, Switzerland; 2 ENT, Lugano, Switzerland; 3 Universitätsklinik für Dermatologie, Inselspital, Universitätsspital Bern, Freiburgstrasse 34, CH-3010 Bern, Switzerland

**Keywords:** Herpes zoster, Trigeminal nerve, Multidermatomal involvement

## Abstract

**Background:**

Herpes zoster, also known as shingles, results from reactivation of the varicella-zoster virus. It commonly presents with burning pain and vesicular lesions with unilateral distribution and affects the thoracic and cervical sites in up to 60 and 20% of cases, respectively. The branches of the trigeminal nerves are affected in up to 20% of cases. Multidermatomal involvement of the trigeminal nerves has been only anecdotally described in immunocompetent subjects.

**Case presentation:**

A 71-year-old previously healthy male presented with grouped vesicular and impetiginized lesions with crusts on the left half of the face of two-weeks duration. The lesions first developed on the left nasal tip and progressively worsened with unilateral appearance of vesicular lesions on the left forehead, face, ala nasi, nasal vestibulum and columella, as well as on the left side of hard and soft palate. The affected edematous erythematous areas corresponded to the distribution of the left ophthalmic (V1) and maxillary (V2) branches of the trigeminal nerve, including the *infraorbital and nasopalatine nerves* of the maxillary branch responsible for the oral cavity involvement. Viral DNA amplification by polymerase chain reaction confirmed the presence of Varicella zoster virus. The patient was started on oral valaciclovir with rapid recovery.

**Conclusions:**

Among immunocompetent patients, herpes zoster is considered a self-limited localized infection. Our observation provides a rare but paradigmatic example of herpes zoster with involvement of both the ophthalmic and maxillary divisions of the trigeminal nerve in an immunocompetent patient. Immunocompetence status and age-specific screening should be warranted in case of atypical involvement and according to the patient’s history, while treatment with antiviral drugs should be rapidily initiated in patients at risk.

## Background

Herpes zoster (HZ), also known as shingles, results from reactivation of the varicella-zoster virus (VZV). It is estimated that there are approximately 1 million new cases per year in the USA [1]. It commonly presents with burning pain and vesicular lesions with unilateral distribution. HZ commonly affects the thoracic and cervical sites in up to 60 and 20% of cases, respectively. Furthermore, the branches of the trigeminal nerves are involved in up to 20% of cases [1, 2]. Multidermatomal involvement of the trigeminal nerves has been rarely described in immunocompetent subjects [1–4]. We here present a striking case of HZ with involvement of ophthalmic and maxillary branches of the fifth cranial nerve in an elderly patient.

## Case presentation

**Fig. 1:**
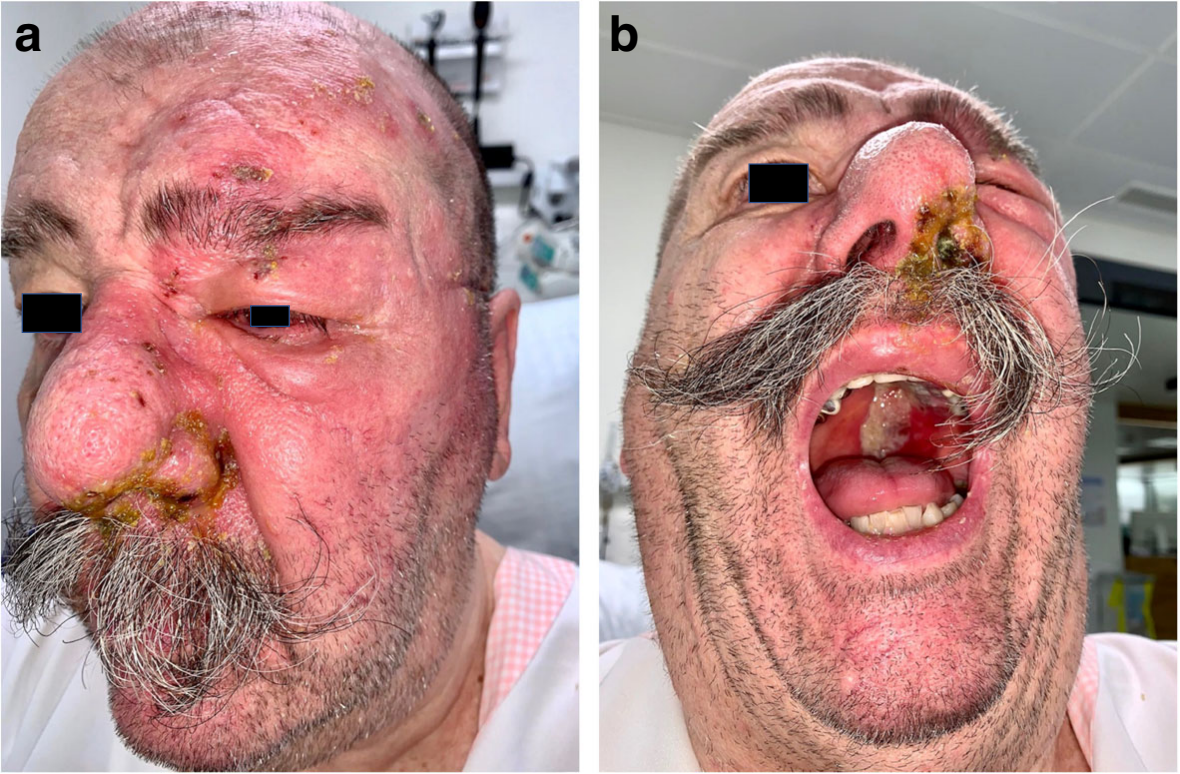
**a** Herpes zoster affecting the left ophthalmic and maxillary divisions of the left trigeminal nerve with erythematous vesicular and impetiginized lesions with crusts on the forehead, the periorbital area, the eye, the ala nasi and upper lip. **b** Involvement of the left vestibulum nasi and columella with unilateral lesions affecting the oral cavity, including the hard and soft palate

A 71-year-old male presented with grouped vesicular lesions with crusts on the left half of the face of two-weeks duration. His past medical history was unremarkable. The lesions first developed on the left nasal tip and progressively worsened with unilateral appearance of new maculopapular and vesicular lesions on the left forehead, face, ala nasi, nasal vestibulum and columella, as well as on the left side of hard and soft palate (Fig. 1a, b). The affected edematous areas corresponded to the distribution of the left ophthalmic (V1) and maxillary (V2) branches of the trigeminal nerve, including the *infraorbital and nasopalatine nerves* of the maxillary branch responsible for the oral cavity involvement.

Ophthalmic examination revealed conjunctival congestion in the left eye with lid edema, but no evidence for herpetic keratitis or uveitis. Viral DNA amplification by polymerase chain reaction (PCR) using smears from forehead and oral mucosa confirmed the presence of VZV, whereas search of Herpes simplex virus type 1 and Herpes simplex virus type 2 by PCR was negative. The rest of the physical examination was normal. Complete blood count, serum electrolytes, hepatic and renal tests were within normal limits. Serological tests for human immunodeficiency virus were negative. The patient subsequently underwent a neck-chest-abdomen-pelvis CT scan, which showed no significant pathologies.

The patient was started on oral valaciclovir 1 g three times a day, oral paracetamol and topical fucidic acid ointments. Hyaluronic acid eye drops 6–8 times in a day were also prescribed. The patient showed complete recovery within 10 days.

## Discussion and conclusions

HZ constitutes a reactivation of a latent infection of VZV, an alpha herpes virus [1–7]. VZV, which infects almost 99.5% of the population of more than 40 years of age has gained access to cranial nerve or dorsal root ganglia during varicella in childhood [1]. The life-time risk of developing HZ is approximately 30% [1]. While reactivation of VZV is normally suppressed by a competent immune system [3], risk factors for HZ include aging, psychological stress, immunosuppression such as cancer treatment, direct trauma, surgery and sunburn [1, 4–6].

Pain and paresthesia in the involved dermatomes occur usually 2–4 days prior to the development of closely grouped erythematous maculo-papules, which then quickly evolve to vesicular and often impetiginized lesions in areas restricted to the sensitive nerve territory of a *dermatome,* including mucosal sites as in our case.

Among immunocompetent patients, HZ is considered a self-limited localized infection. Nevertheless, it may cause significant morbidity and complications. Severe neurological complications, including postherpetic neuralgia (occurs in approximately 20% of cases), sensory loss and palsy, as well as ocular, cutaneous and visceral complications with viremia may variably occur [1, 5, 8–10]. In contrast to immunodeficient patients, multidermatomal involvement in HZ is rarely observed in immunocompetent patients. The 1st ophthalmic branch of the fifth cranial nerve is affected about 20 times more often than the 2nd and 3rd branches of the nerve [8].

Noteworthy, only anecdotal reports exist about HZ affecting two or three branches of the trigeminal nerve [2–4, 6, 9]. In our *PubMed search* using terms “herpes zoster”, “multidermatomal”, “trigeminal nerve” and “ophthalmicus” we have only identified 5 reports describing cases with HZ affecting two or all three branches of the trigeminal nerve (Table 1). Although multidermatomal involvement of the trigeminal nerve branches during HZ infection has been only very rarely reported in the literature, it is important that clinicians are aware and more familiar with this presentation, which seems not so rare according to specialists who have been practicing long enough.

**Table 1:** Summary of published cases of multidermatomal herpes zoster affecting the trigeminal nerve

Authors	Patient age/sex	Affected dermatomes	Comorbidities
Dube et al. [2]	55/M	V1–3	None reported
Lovell [3]	57/F	V1–2	Rheumatoid arthritis
Nair et al. [4]	68/M and 50/M	V2–3 and V1–3	None reported
Srivastava et al. [6]	55/F	V1–2	None reported
Naveen et al. [9]	28/M	V1–3	None reported

Practically, in case of ophthalmic zoster, the eye is directly involved in 50–72% of patients potentially leading to visual impairment, ptosis, pain, keratitis and facial scarring [2, 5]. In our case, only a conjunctival congestion was present. Presence of lesions on the tip or side of the nose indicate involvement of nasociliary nerve of the ophthalmic branch of the trigeminal nerve, known as Hutchinson’s sign [5]. The latter thus warrants a close ophthalmologic evaluation to exclude subsequent ocular involvement. Reactivation of VZV within the maxillary (V2) division may lead to pain and lesions affecting upper lip as well as on the soft and hard palate, while involvement of the mandibular (V3) nerve is associated with manifestations affecting the anterior part of the tongue, floor of oral cavity and buccal mucous membrane [9].

Our observation provides a paradigmatic example of HZ with involvement of both the ophthalmic and maxillary divisions of the trigeminal nerve in an immunocompetent elderly patient in good general health.

In case of atypical presentations as well as in immunosuppressed patients laboratory testing is indicated to confirm the diagnosis of VZV infection. Immunofluorescence microscopy studies for detection of VZV antigens in tissue smears, viral culture or PCR for VZV DNA assays are useful according to the clinical presentation and practical availability [9].

Different oral antivirals, including valaciclovir, aciclovir, famciclovir and brivudine currently constitute the first therapeutic option based on specific indications and associated comorbidities [10]. Our patient was given oral valaciclovir with complete rapid resolution of the lesions. Nevertheless, in HZ with involvement of the head with either complicated infection or at risk for a complicated course intravenous aciclovir is recommended [10].

Immunocompetence status and age-specific screening should be warranted in case of atypical involvement and according to the patient’s history, while treatment with antiviral drugs should be rapidly initiated in patients at risk for complications.

## Data Availability

Data sharing is not applicable to this article as no datasets were generated or analyzed during the current study.

## Abbreviations

HZ: Herpes zoster
VZV: Varicella zoster virus
PCR: Polymerase chain reaction
